# Validity Assessment of 5 Day Repeated Forced-Swim Stress to Model Human Depression in Young-Adult C57BL/6J and BALB/cJ Mice

**DOI:** 10.1523/ENEURO.0201-16.2016

**Published:** 2016-12-29

**Authors:** Joram D. Mul, Jia Zheng, Laurie J. Goodyear

**Affiliations:** 1 Section on Integrative Physiology and Metabolism, Joslin Diabetes Center, Harvard Medical School, Boston, Massachusetts 02215; 2 Department of Medicine, Brigham, and Women’s Hospital, Harvard Medical School, Boston, Massachusetts 02215

**Keywords:** anhedonia, animal model, depression, forced swimming, stress, voluntary wheel running

## Abstract

The development of animal models with construct, face, and predictive validity to accurately model human depression has been a major challenge. One proposed rodent model is the 5 d repeated forced swim stress (5d-RFSS) paradigm, which progressively increases floating during individual swim sessions. The onset and persistence of this floating behavior has been anthropomorphically characterized as a measure of depression. This interpretation has been under debate because a progressive increase in floating over time may reflect an adaptive learned behavioral response promoting survival, and not depression (Molendijk and de Kloet, 2015). To assess construct and face validity, we applied 5d-RFSS to C57BL/6J and BALB/cJ mice, two mouse strains commonly used in neuropsychiatric research, and measured a combination of emotional, homeostatic, and psychomotor symptoms indicative of a depressive-like state. We also compared the efficacy of 5d-RFSS and chronic social defeat stress (CSDS), a validated depression model, to induce a depressive-like state in C57BL/6J mice. In both strains, 5d-RFSS progressively increased floating behavior that persisted for at least 4 weeks. 5d-RFSS did not alter sucrose preference, body weight, appetite, locomotor activity, anxiety-like behavior, or immobility behavior during a tail-suspension test compared with nonstressed controls. In contrast, CSDS altered several of these parameters, suggesting a depressive-like state. Finally, predictive validity was assessed using voluntary wheel running (VWR), a known antidepressant intervention. Four weeks of VWR after 5d-RFSS normalized floating behavior toward nonstressed levels. These observations suggest that 5d-RFSS has no construct or face validity but might have predictive validity to model human depression.

## Visual Abstract

**Figure:**
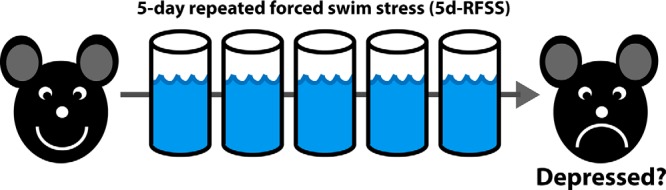

## Significance Statement

The development of valid animal models to model human depression has been a major challenge. One protocol that has been widely used for its presumptive effects to cause depression in mice is the 5 d repeated forced swim stress (5d-RFSS) paradigm. 5d-RFSS increases floating behavior during consecutive sessions, but whether this is depressive-like behavior or an adaptive response underlying survival is not clear. We subjected two mouse strains (C57BL/6J, BALB/cJ) to 5d-RFSS followed by a battery of reward-related, homeostatic, and behavioral tests. 5d-RFSS increased floating behavior over time but, importantly, did not induce emotional, homeostatic, or psychomotor symptoms. These findings suggest that 5d-RFSS has no construct or face validity to model human depression in two mouse strains commonly used in neuropsychiatric research.

## Introduction

Major depressive disorder affects approximately one in six individuals during their lifetime and has an enormous social and financial impact on modern society (Greenberg et al., 2003; Kessler et al., 2005). This psychiatric disorder is diagnosed based on symptoms with considerable heterogeneity and without known highly penetrant genetic causes (Krishnan and Nestler, 2008). There is a general need for animal models to study the pathophysiology of depression and identify therapeutic interventions. However, the development of animal models of depression with construct, face, and predictive validity has been a major challenge (Nestler and Hyman, 2010; Belzung and Lemoine, 2011). Nevertheless, several ethologically valid rodent models of depression have been developed and validated, including chronic unpredictable stress (CUS) and chronic social defeat stress (CSDS) (Willner, 2005; Nestler and Hyman, 2010). Despite the validity of these animal models, lack of ethical approval or other limitations has stimulated investigators to keep exploring additional rodent paradigms that model human depression.

One proposed animal model is the 5 d repeated forced swim stress (5d-RFSS) paradigm, during which mice are forced to swim in a beaker filled with water for 10 min over 5 consecutive days. Several studies have reported a progressive increase in floating during consecutive forced swimming sessions that was maintained for at least 4 weeks, and this behavior is commonly interpreted as the onset and persistence of a depressive-like state (Stone and Lin, 2011; Sun et al., 2011; Serchov et al., 2015). This interpretation has been debated, as increased floating behavior during repeated forced swimming sessions may rather reflect an adaptive learned behavioral response underlying survival (Molendijk and de Kloet, 2015). It has been suggested that to provide more definitive evidence on the presence of a depressive-like state, a combination of emotional symptoms (anhedonia), homeostatic symptoms (sleep, appetite, body weight), psychomotor symptoms (locomotor activity, immobility- and anxiety-like behavior), or direct assessment of the reward circuitry of the brain should be measured (Nestler and Hyman, 2010). Emotional, homeostatic, and psychomotor symptoms are hallmarks of human depression and are important parameters that can be measured objectively in rodents (Nestler and Hyman, 2010).

5d-RFSS in mice was recently shown to decrease sucrose preference immediately following 5d-RFSS, suggesting anhedonia, and this emotional symptom persisted for at least 4 weeks (Serchov et al., 2015). Maintenance of anhedonia allows for a time window to test and study therapeutic interventions. Here we determined the validity of 5d-RFSS to model human depression using a battery of tests that assessed emotional, homeostatic, and psychomotor symptoms. In other words, does 5d-RFSS induce a depressive-like state in mice? For this purpose, we applied a similar 5d-RFSS protocol, as used by Serchov et al. (2015), to two inbred strains commonly used in neuropsychiatric research: young-adult, male, stress-resilient C57BL/6J mice and stress-susceptible BALB/cJ mice (Pothion et al., 2004; Farley et al., 2010; Razzoli et al., 2011; Serchov et al., 2015). To thoroughly assess the onset of a depressive-like state, we measured sucrose preference, body weight, food intake, locomotor activity, anxiety-like behavior, and immobility behavior during a tail-suspension test (TST) before and after 5d-RFSS. We also compared the efficacy of 5d-RFSS and CSDS, a validated model of depression (Kudryavtseva et al., 1991; Krishnan et al., 2007; Nestler and Hyman, 2010; Golden et al., 2011), to induce a depressive-like state in C57BL/6J mice.

Predictive validity relies on the observation that treatment modalities effective in reversing depression in humans should reverse the changes observed in an animal model of depression (McKinney and Bunney, 1969; Belzung and Lemoine, 2011). In line with this, several studies have demonstrated that known antidepressant drugs (imipramine, ketamine, fluoxetine, tranylcypromine) or treatments [repetitive transcranial magnetic stimulation (rTMS)] decrease the persistence of increased floating behavior induced by 5d-RFSS (Stone and Lin, 2011; Sun et al., 2011; Serchov et al., 2015), thus suggesting predictive validity. Therefore, we also investigated whether voluntary wheel running (VWR), a behavioral intervention that mimics human exercise training and has antidepressant action (North et al., 1990; Brené et al., 2007), modulates the persistence of increased floating behavior induced by 5d-RFSS.

## Material and Methods

### Animals

Experiments were conducted in accordance with the Joslin Diabetes Center Institutional Animal Care and Use Committee. Ten-week old male C57BL/6J (https://www.jax.org/strain/000664) and BALB/cJ (https://www.jax.org/strain/000651) mice were group housed and habituated to the Joslin Diabetes Center animal facility for at least 2 d before the onset of experimental treatments. This time frame was sufficient for body weights to return to stable pretravel levels (data not shown). Mice were maintained at 23–25°C on a 12 h light/dark cycle (lights on from 6:30 A.M.) with *ad libitum* access to a pelleted chow diet [9F 5020 Lab Diet (23% protein, 55% carbohydrate, and 22% fat, 3.56 kcal/g), PharmaServ] and water, unless noted otherwise. All experimental groups were body weight matched at the onset of the experiments. Body weight and available food were measured at indicated time points. All behavioral assessments, except for the two-bottle sucrose preference tests (SPTs), were performed in an experimental room, and mice were acclimatized to the experimental room for 2 h before the start of behavioral studies.

### The 5 d repeated forced-swim stress paradigm

**Figure 1.**
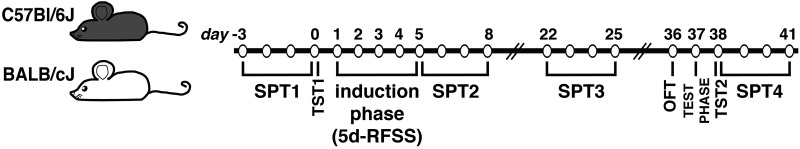
5d-RFSS paradigm. Experimental timeline of 5d-RFSS in C57BL/6J or BALB/cJ mice. SPT1 and TST1 were performed on days −3 to 0, before stressed mice underwent 10 min of forced-swim stress during 5 consecutive days (days 1–5; induction phase), followed by SPT2 (days 5–8), SPT3 (days 22–25), an OFT (day 36), the last forced swim session (test phase; day 37), TST2 (day 38), and SPT4 (days 38–41). Nonstressed controls were not forced to swim during the induction phase but otherwise underwent the same protocol.

All experimental mice were single housed from the start of the 5d-RFSS (Fig. 1) paradigm. We used a recently described 5d-RFSS protocol (Serchov et al., 2015) with one modification: addition of an extra SPT (i.e., SPT3) during days 22–25. Stressed mice were forced to swim in an open cylindrical container (diameter, 12 cm; height, 28 cm) containing 19 cm of water (25 ± 1°C) on 5 consecutive days (days 1–5; induction phase) and on day 37 (test phase; Fig. 1). Individual tests lasting 10 min were monitored from the top and scored automatically using the ANY-maze software (version 4.98; Stoelting). Immobile behavior sensitivity was set at 65%, and the mouse needed to be immobile for 500 ms to initiate the scoring of immobility. In general, each mouse was judged to be immobile when it ceased struggling and remained floating motionless in the water, making only movements necessary to keep its head above the water surface (Duman et al., 2008; Cunha et al., 2013). Water was changed between each test. Nonstressed controls were physically handled briefly by the investigator, transferred to a transport cup for 10 min, and returned to their home cage.

### SPT

Mice were given free access to two drinking pipettes in their home cage, one containing 1% (C57BL6/J) or 3% (BALB/cJ) sucrose solution and the other containing water. Fluid consumption was measured in the early afternoon, and the position of the pipettes was interchanged daily to prevent a place preference. Sucrose preference is calculated as the percentage of the amount of sucrose solution consumed over total fluid consumption ([sucrose solution intake/total fluid intake] × 100) and was averaged over all 3 d of testing.

### TST

Mice were suspended by adhesive tape placed ∼1 cm from the tip of their tail that was taped to a horizontal holder so that the mouse was suspended 20 cm above a horizontal surface. The mouse tail was passed through a small plastic cylinder prior to suspension to prevent tail-climbing behavior. Individual tests lasting 6 min were monitored and scored automatically using the ANY-maze software (version 4.98; Stoelting). Immobile behavior sensitivity was set at 70%, and the mouse needed to be immobile for 1 s to initiate scoring of immobility. After the TST, mice were returned to their respective home cages.

### Open-field test

During the light phase, mice were placed in a rectangular open field monitoring setup (59 × 29 cm; walls, 30 cm; nonreflecting gray PVC), and locomotor activity was monitored from the top for 6 min and scored automatically using the ANY-maze software (version 4.98; Stoelting). The center zone was defined as a 20 × 10 cm zone designated in the middle of the open field. Feces produced during the open-field test (OFT) were counted manually. Mice were immediately returned to their respective home cages after the OFT.

### Chronic social defeat stress and behavioral evaluations

CSDS was performed as described with a few modifications (Krishnan et al., 2007; Vialou et al., 2010; Golden et al., 2011). In short, all mice were tested during SPT1 (day −3 to 0) before the start of 10 consecutive days of CSDS. During each defeat episode, experimental C57BL/6J mice (intruder) were allowed to interact for 10 min with an unfamiliar CD1 aggressor (resident), during which they displayed subordinate posturing. Intruders then spent the remainder of each 24 h period in the cage of the aggressor, separated from the aggressor by a custom-made perforated aluminum partition (sensory housing). Undefeated C57BL/6J controls were housed by pair, one on each side of a perforated aluminum partition (no physical interaction), and were handled daily. For the social interaction test on day 11, time spent in the interaction zone during the first (target absent) and second (social target present) 2.5 min trials were automatically scored using ANY-maze software (version 4.98; Stoelting). Unfamiliar CD1 mice that did not partake in the defeat episodes were used as social targets. Following the social interaction test, all mice were tested during an OFT (day 12) and during SPT2 (days 12–15).

### Voluntary wheel running

Mice were housed without a running wheel (sedentary; SED) or were given voluntary access to an active running wheel (VWR; 24 cm diameter; Nalgene) for 4 weeks. Wheel revolutions were measured daily using odometers.

### Statistical analysis

Data are displayed as the mean ± SEM. For all experiments, single comparisons between means were analyzed by unpaired *t* test, whereas SPT1 and SPT2 were compared using a paired *t* test. Multiple comparisons between means were analyzed using one- or two-way ANOVA, with repeated measures where applicable. Daily VWR distances were analyzed using one-way ANOVA, with repeated measures. If appropriate, *post hoc* analyses were made using a Tukey’s HSD test, with *p* < 0.05 accepted as being statistically different.

## Results

### Effects of 5d-RFSS on depressive-like behavior in C57BL/6J mice

**Figure 2.**
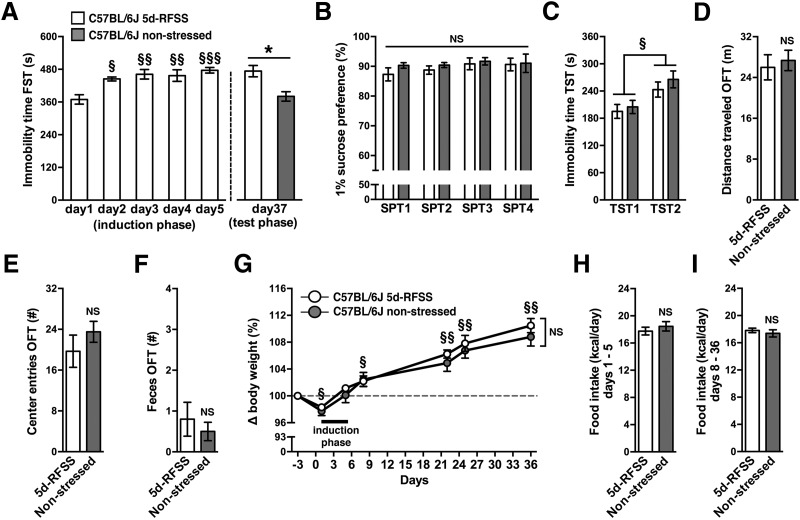
Effects of 5d-RFSS on depressive-like behavior in C57BL/6J mice. ***A***, Immobility time of 5d-RFSS C57BL/6J mice during the induction phase (days 1–5) forced-swim tests (FST; main effect of time, *F*_(4,36)_ = 8.68, *p* = 0.00005; *post hoc* test, §*p* = 0.006, §§*p* = 0.001, §§§*p* = 0.0002 vs day 1), and of 5d-RFSS and nonstressed control mice during the test phase (day 37; *t*_(1,18)_ = 3.44, *p = 0.003 vs nonstressed mice). ***B***, Two-bottle SPTs before 5d-RFSS (SPT1) and after 5d-RFSS (SPT2 to SPT4). ***C***, TST immobility time before 5d-RFSS (TST1) and after 5d-RFSS (TST2; main effect of time, *F*_(1,18)_ = 9.14, *p* = 0.007; *post hoc* test, §*p* = 0.007 vs TST1). ***D–F***, Distance traveled (*t*_(1,18)_ = 0.43, *p* = 0.67; ***D***), number of center zone entries (*t*_(1,18)_ = 1.01, *p* = 0.33; ***E***), and number of feces produced during the OFT (*t*_(1,18)_ = 0.63, *p* = 0.53; ***F***). ***G***, Change in body weight (%) from day −3 to 36 (main effect of time, *F*_(6,108)_ = 90.48, *p* < 0.00001; *post hoc* test, §*p* = 0.02, §§*p* = 0.0001 vs day −3). ***H***, ***I***, Food intake during induction phase (days 1–5; *t*_(1,17)_ = 0.77, *p* = 0.45; ***H***) and during days 8–36 (*t*_(1,18)_ = 0.71, *p* = 0.49; ***I***). NS, Not significant. *n* = 10/group for all experiments.

We first tested the hypothesis that 5d-RFSS induces a depressive-like state in C57BL/6J mice using a previously described 5d-RFSS protocol (Serchov et al., 2015). In this paradigm, single-housed mice are stressed by being forced to swim on 5 consecutive days (i.e., 5d-RFSS; induction phase) and depressive-like behavior was assessed using a battery of behavioral tests before and after the induction phase (Fig. 1). Nonstressed C57BL/6J controls were handled daily during the induction phase but were not forced to swim. C57BL/6J mice that underwent 5d-RFSS demonstrated a progressive increase in floating behavior during the induction phase (Fig. 2*A*). During the test phase 32 d later, mice that had undergone 5d-RFSS continued to demonstrate relatively high levels of floating behavior (Fig. 2*A*). In line with this persistence, floating behavior scores were significantly greater than those of nonstressed C57BL/6J controls that had not been forced to swim during the induction phase (Fig. 2*A*). When given a choice between water and 1% sucrose before undergoing 5d-RFSS (SPT1), C57BL/6J mice showed a typical and strong preference for sucrose (∼88%; Fig. 2*B*). None of the 5d-RFSS mice developed a loss of sucrose preference (i.e., anhedonia) compared with nonstressed C57BL/6J controls or compared with pre-5d-RFSS (SPT1) levels (Fig. 2*B*). All experimental mice demonstrated greater immobility scores during TST2 compared with TST1, independent of having been forced to swim (Fig. 2*C*). Immobility behavior during the test-phase swim session on day 37 or during TST2 was not associated with altered general locomotor activity as indicated by similar distance traveled during an OFT (Fig. 2*D*). The number of center zone entries or feces produced during the OFT, both of which are parameters indicative of anxiolytic behavior, also did not differ between experimental groups (Fig. 2*E*,*F*). Similarly, time spent in the center zone did not differ between nonstressed C57BL/6J controls and mice that underwent the 5d-RFSS (38.8 ± 4.7 vs 34.6 ± 4.6 s, respectively; *t*_(1,18)_ = 0.64, *p* = 0.53). Five consecutive days of forced swimming had no immediate effect on body weight in 5d-RFSS mice compared with controls that were handled daily but were not forced to swim (Fig. 2*G*). All C57BL/6J mice showed similar increases in body weight during the 4 weeks following 5d-RFSS, independent of having been forced to swim (Fig. 2*G*). In contrast, switching from group housing to single housing at the start of the 5d-RFSS paradigm slightly lowered body weight in all C57BL/6J mice (Fig. 2*G*). 5d-RFSS did not alter caloric intake in the short term during the 5d-RFSS (days 1–5) or in the 4 weeks following 5d-RFSS (days 8–36; Fig. 2*H*,*I*).

### Effects of 5d-RFSS on depressive-like behavior in BALB/cJ mice

**Figure 3.**
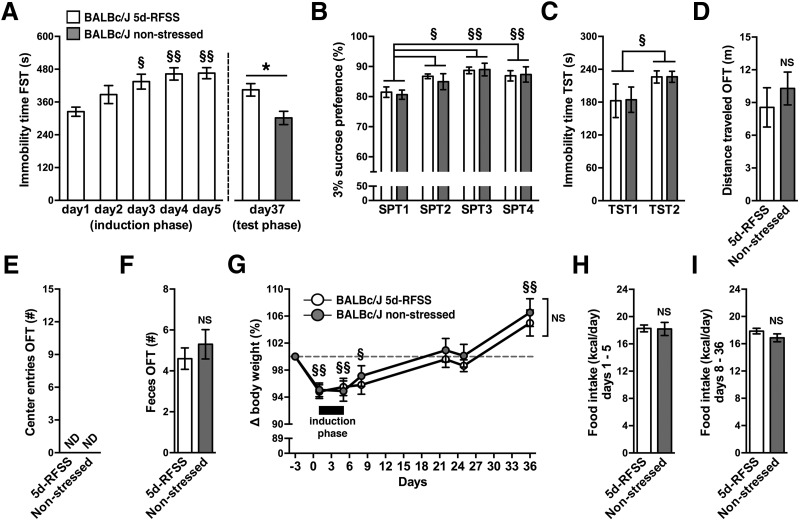
Effects of 5d-RFSS on depressive-like behavior in BALB/cJ mice. ***A***, Immobility time of 5d-RFSS BALB/cJ mice during the induction phase (days 1–5) of forced swim tests (FST; main effect of time, *F*_(4,36)_ = 6.29, *p* = 0.0006; *post hoc* test, §*p* = 0.02, §§*p* = 0.002 vs day 1) and of 5d-RFSS and nonstressed control mice during the test phase (day 37; *t*_(1,18)_ = 3.08, *p = 0.007). ***B***, Two-bottle SPTs before 5d-RFSS (SPT1) and after 5d-RFSS (SPT2 to SPT4; main effect of time, *F*_(3,54)_ = 12.67, *p* < 0.00001; *post hoc* test, §*p* = 0.002, §§*p* = 0.0002 vs SPT1). ***C***, TST immobility time before 5d-RFSS (TST1) and after 5d-RFSS (TST2; main effect of time, *F*_(1,18)_ = 5.06, *p* = 0.04; *post hoc* test, §*p* = 0.04 vs TST1). ***D–F***, Distance traveled (*t*_(1,18)_ = 0.74, *p* = 0.47; ***D***), number of center zone entries (***E***), and number of feces produced during the OFT (*t*_(1,18)_ = 0.79, *p* = 0.44; ***F***). ***G***, Change in body weight (%) from days −3 to 36 (main effect of time, *F*_(6,108)_ = 36.96, *p* < 0.00001; *post hoc* test, §*p* = 0.002, §§*p* = 0.0001 vs day −3). ***H***, ***I***, Food intake during the induction phase (days 1 – 5; *t*_(1,18)_ = 0.07, *p* = 0.94; ***H***) and during days 8–36 (*t*_(1,18)_ = 1.36, *p* = 0.19; ***I***). NS, Not significant; ND, not detected. *n* = 10/group for all experiments.

We next tested the hypothesis that 5d-RFSS induces a depressive-like state in BALB/cJ mice. This mouse strain, compared with C57BL/6J mice, is particularly sensitive to the development of stress-induced anhedonia (Farley et al., 2010; Razzoli et al., 2011). Because our preliminary data indicated that BALB/cJ mice do not generate a sucrose preference to a 1% sucrose solution (data not shown), we used a 3% sucrose solution with this strain. BALB/cJ mice that underwent 5d-RFSS demonstrated a progressive increase in floating behavior during the induction phase (Fig. 3*A*). Nonstressed BALB/cJ controls were handled daily during the induction phase but were not forced to swim. During the test phase 32 d later, mice that had undergone 5d-RFSS continued to demonstrate increased floating behavior (Fig. 3*A*). In line with this persistence, floating behavior scores were significantly greater than those in nonstressed BALB/cJ controls that had not been forced to swim during the induction phase (Fig. 3*A*). When given a choice between water and 3% sucrose before 5d-RFSS (SPT1), BALB/cJ mice showed a typical and strong preference for sucrose (∼81%; Fig. 3*B*). None of the 5d-RFSS mice developed a loss of sucrose preference (i.e., anhedonia) compared with nonstressed BALB/cJ controls or with pre-5d-RFSS (SPT1) levels (Fig. 3*B*). Sucrose preference was actually slightly higher during SPT2 to SPT4, independent of the treatment of the mice (Fig. 3*B*). BALB/cJ mice demonstrated greater immobility scores during TST2 compared with TST1, independent of having been exposed to swim stress (Fig. 3*C*). Immobility behavior during the test phase swim session on day 37 or during TST2 was not associated with altered general locomotor activity, as indicated by the similar distance traveled during an OFT (Fig. 3*D*). None of the BALB/cJ mice entered the center zone during the OFT (Fig. 3*E*). The number of feces produced during the OFT did not differ between 5d-RFSS mice and nonstressed controls (Fig. 3*F*). Similar to the C57BL/6J cohort, 5 consecutive days of forced swimming had no immediate effect on body weight in 5d-RFSS mice compared with controls that were handled daily but were not forced to swim (Fig. 3*G*). Furthermore, all BALB/cJ mice showed similar increases in body weight during the 4 weeks following 5d-RFSS, independent of having been forced to swim (Fig. 3*G*). In contrast, switching from group housing to single housing at the start of the 5d-RFSS paradigm slightly lowered body weight in all BALB/cJ mice (Fig. 3*G*). 5d-RFSS did not alter caloric intake acutely during 5d-RFSS (days 1–5) or in the 4 weeks following 5d-RFSS (days 8–36; Fig. 3*H*,*I*).

### CSDS induces a depressive-like state in C57BL/6J mice

**Figure 4.**
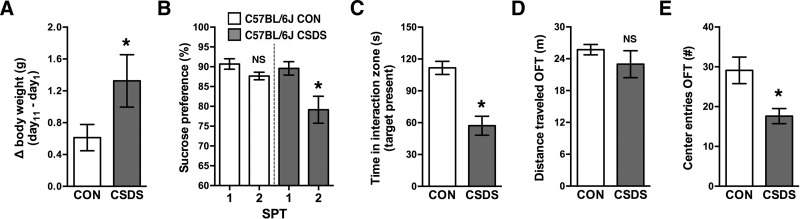
CSDS induces a depressive-like state in C57BL/6J mice. ***A***, CSDS mice had greater weight gain compared with nondefeated control (CON) mice (days 1 – 11; *t*_(1,14)_ = 2.28, *p = 0.039). ***B***, CON mice did not show a significant decrease in sucrose preference during two-bottle SPT2 compared with SPT1 (*t*_(1,7)_ = 1.96, *p* = 0.1). CSDS mice demonstrated lower sucrose preference during SPT2 compared with SPT1 (before CSDS; *t*_(1,7)_ = 3.16, *p = 0.016). ***C***, CSDS mice spent less time in the interaction zone with target present during the social interaction test on day 11 (*t*_(1,14)_ = 5.04, *p = 0.00018). ***D***, Locomotor activity did not differ significantly between experimental groups during OFT on day 12 (*t*_(1,14)_ = 1.01, *p* = 0.33). ***E***, CSDS mice enter OFT center zone less than CON mice (*t*_(1,14)_ = 2.99, *p = 0.01). NS, Not significant. *n* = 8/group for all experiments.

CSDS is a validated model of depression in C57BL/6J mice (Kudryavtseva et al., 1991; Krishnan et al., 2007; Nestler and Hyman, 2010; Golden et al., 2011). Therefore, we next used this animal model as a comparison to determine our ability to induce a depressive-like state in young-adult male C57BL/6J mice. Mice that had undergone 10 d of social defeat stress had greater body weight gain compared with nondefeated controls (Fig. 4*A*). CSDS mice showed a significant decrease in sucrose preference during SPT2 compared with pre-CSDS levels (SPT1), whereas nondefeated controls did not (Fig. 4*B*). As a second parameter of depressive-like behavior, we also measured social avoidance following CSDS (Krishnan et al., 2007). When tested during a social interaction test on day 11, CSDS mice spent less time interacting with a social target than nondefeated controls (Fig. 4*C*). Finally, CSDS mice had fewer center zone entries during an OFT, which is indicative of increased anxiety-like behavior, and this was independent of total locomotor activity during the OFT (Fig. 4*D*,*E*).

### Effects of VWR on persistence of immobility behavior following 5d-RFSS

**Figure 5.**
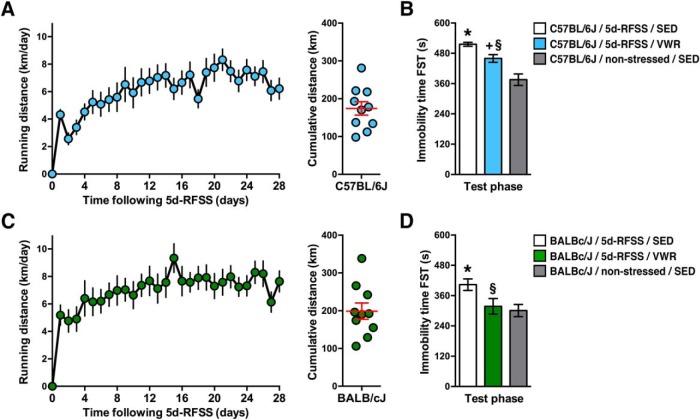
Effects of VWR on the persistence of immobility behavior following 5d-RFSS. ***A***, Daily VWR distance (left; *F*_(27,243)_ = 5.89, *p* < 0.00001) and cumulative VWR distance (right) of C57BL/6J mice. ***B***, VWR (28 d) following 5d-RFSS lowered immobility scores during the test day swim session toward levels of nonstressed SED mice (*F*_(2,27)_ = 18.45, *p* = 0.00001; *post hoc* test, *p = 0.00013 vs nonstressed SED mice; +*p* = 0.0034 vs nonstressed SED; §*p* = 0.059 vs 5d-RFSS SED mice). ***C***, Daily VWR distance (left; *F*_(27,243)_ = 4.37; *p* < 0.00001) and cumulative VWR distance (right) of BALBc/J mice. ***D***, VWR (28 d) following 5d-RFSS normalized immobility scores during the test day swim session to levels of nonstressed SED mice (*F*_(2,27)_ = 4.41, *p* = 0.02; *post hoc* test, *p = 0.026 vs nonstressed SED, §*p* = 0.068 vs 5d-RFSS SED). *n* = 10/group for all experiments.

Antidepressant drugs and treatments decrease the persistence of increased floating behavior induced by 5d-RFSS (Stone and Lin, 2011; Sun et al., 2011; Serchov et al., 2015), which suggests predictive validity. Therefore, we tested whether VWR, another known antidepressant intervention (North et al., 1990), could modulate the persistence of immobility behavior induced by 5d-RFSS. Following 5d-RFSS, C57BL/6J mice were given voluntary access to running wheels for 28 d (Fig. 5*A*,*B*). VWR after 5d-RFSS lowered the relatively high levels of floating behavior toward nonstressed C57BL/6J control levels (Fig. 5*C*). In BALB/cJ mice, VWR was even more effective and fully normalized floating behavior to nonstressed BALB/cJ control levels (Fig. 5*C*,*D*).

## Discussion

We assessed whether 5d-RFSS has construct, face, and predictive validity to model human depression by determining whether 5d-RFSS induces a depressive-like state in C57BL/6J and BALB/cJ mice. These inbred strains, obtained from a commercial breeder, are commonly used in neuropsychiatric research and have relatively low and high emotionality, respectively (Farley et al., 2010; Razzoli et al., 2011). We thoroughly assessed depressive-like behavior before and after 5d-RFSS by measuring sucrose preference, body weight, food intake, TST immobility behavior, and anxiety-like behavior. Our observations suggest that 5d-RFSS induces an adaptive learned behavioral response, but not a depressive-like state in two mouse strains commonly used in neuropsychiatric research. These findings indicate that 5d-RFSS has no construct or face validity to model human depression. However, because the persistence of the adaptive and learned behavioral response is modulated by known antidepressant drugs (Stone and Lin, 2011; Serchov et al., 2015), rTMS (Sun et al., 2011), and VWR (this study), 5d-RFSS might have predictive validity.

In line with previous reports (Sun et al., 2011; Serchov et al., 2015), 5d-RFSS induced a progressive increase in floating during individual swim sessions in our C57BL/6J and BALB/cJ cohorts, and this increase in floating behavior persisted for at least 4 weeks. Our observations thus confirm that repeated forced-swim sessions can induce an adaptive and learned behavioral response underlying survival in mice (Molendijk and de Kloet, 2015).

C57BL/6J or BALB/cJ mice did not develop loss of sucrose preference (i.e., anhedonia) immediately following 5d-RFSS or during the 4 weeks following 5d-RFSS. In contrast, it has been reported (Serchov et al., 2015) that 5d-RFSS induced anhedonia immediately after 5d-RFSS, and this anhedonia could be rescued following 4 weeks of enhanced adenosine A_1_ receptor expression in the brain. Although experimental differences are potential explanations for the contrasting observations, it is difficult to directly compare our findings, as this study did not specify the exact genetic background, age, or sex of the experimental mice for each individual experiment (Serchov et al., 2015). Our observations suggest that wild-type C57BL/6J and BALB/cJ mice obtained directly from a popular commercial breeder do not develop anhedonia following 5d-RFSS, suggesting that genetic background can have important effects on behavioral responses to stressors. Finally, a genetic model used by Serchov et al. (2015) was raised on doxycycline in their drinking water until weaning, which, as recognized by the authors, makes water extremely bitter. Thus, alterations in drinking behavior or sucrose-sensing deficits during adulthood cannot be excluded in these studies. Exposure to CSDS has revealed susceptible and unsusceptible subpopulations in C57BL/6J mice (Krishnan et al., 2007). In our 5d-RFSS cohorts, all C57BL/6J and BALB/cJ mice demonstrated stable and high (>75%) sucrose preferences, suggesting that it is unlikely that our experimental cohorts contained susceptible and unsusceptible subpopulations. To properly assess sucrose preference, we averaged drinking behavior over 3 d. Importantly, all mice showed stable consumption behavior during each SPT. Moreover, sucrose preference was stable over the 3 consecutive days of SPT1, suggesting that initial reactivity to single housing (conducted on the same day as the start of SPT1) did not contribute significantly.

Changes in body weight or caloric intake are important homeostatic symptoms associated with human depression and can be replicated using established animal models of depression such as CUS and CSDS (Willner, 2005; Nestler and Hyman, 2010). 5d-RFSS did not alter food intake in either strain, both during the 5d-RFSS and in the 4 weeks following the 5d-RFSS. Furthermore, 5d-RFSS had no immediate or delayed effect on body weight compared with nonstressed controls in either strain. In contrast, switching from group housing to single housing induced a transient and small decrease in body weight in all mice at the start of the 5d-RFSS protocol. Social isolation can be a mild-to-severe stressor in mice depending on the duration of social isolation (Wallace et al., 2009), suggesting that short-term isolation by single housing of the experimental mice had a larger effect on body weight than 5d-RFSS. Body weights were analyzed for 4 weeks following 5d-RFSS, suggesting that it is unlikely that body weights will differentiate at a later time point.

All experimental mice demonstrated greater immobility scores during TST2 compared with TST1, again suggesting an adaptive and learned behavioral response. Importantly, TST2 immobility scores were similar between mice that had undergone 5d-RFSS and nonstressed controls. Although the TST is a treatment-based screen with only predictive validity (Nestler and Hyman, 2010), these observations support the notion that 5d-RFSS did not induce depressive-like behavior.

Many stress-based rodent models, including CSDS, also exhibit anxiety-like behavior (Krishnan et al., 2007; Nestler and Hyman, 2010). In contrast, 5d-RFSS did not promote anxiety-like behavior compared with nonstressed controls, as indicated by a similar number of center zone entries, center zone time, or feces produced during the OFT. These observations indicate that 5d-RFSS did not induce the anxiety-like behavior often observed in validated rodent models of depression. BALB/cJ mice produced more feces and showed complete avoidance of the center zone during the OFT compared with C57BL/6J mice, confirming their greater emotionality (Farley et al., 2010; Razzoli et al., 2011).

Together, our observations indicate that 5d-RFSS did not induce emotional, homeostatic, or psychomotor symptoms in young-adult male C57BL/6J and BALB/cJ mice obtained from a commercial breeder. In contrast, CSDS induced changes in body weight, a significant decrease in sucrose preference (i.e., anhedonia), social avoidance, and increased anxiety-like behavior in young-adult male C57BL/6J mice. Collectively, these symptoms are indicative of a depressive-like state (Krishnan et al., 2007; Nestler and Hyman, 2010). Although we cannot exclude that 5d-RFSS can induce a depressive-like state in older mice or in stress-susceptible genetic models, our findings suggest that 5d-RFSS has no construct or face validity to model human depression in two inbred strains commonly used in neuropsychiatric research. However, because known antidepressant treatments, including drugs (Stone and Lin, 2011; Serchov et al., 2015), rTMS (Sun et al., 2011), and VWR (this study), can modulate the persistence of increased floating behavior, 5d-RFSS might have predictive validity to identify novel antidepressant treatments. Finally, the initial assessment of depressive-like behavior in rodents can include treatment-based screens, such as the forced-swim test and TST. However, a combination of emotional, homeostatic, and psychomotor symptoms should be measured to provide more definitive evidence of a depressive-like state.

## Synthesis

Reviewing Editor: Tallie Z. Baram, University of California-Irvine Decisions are customarily a result of the Reviewing Editor and the peer reviewers coming together and discussing their recommendations until a consensus is reached. When revisions are invited, a fact-based synthesis statement explaining their decision and outlining what is needed to prepare a revision will be listed below. The following reviewer(s) agreed to reveal their identity: Victoria Risbrough

The reviewers felt that there was significant merit in the paper:The field needs to reject poor models and findings that are not replicable. These findings are important for the depression field to move towards models that are reliable and offer good validity.

The reviewers also had significant and serious concerns about the paper in its current format. They believed that additional experiments as well as modifications and clarification of reported methodologies and results are required. their comments are enclosed below.

Reviewer 1:

This study attempts to test the question if the 5d-RFSS task has validity as a model of depression. The authors argue, and show, that the immobility observed in this task is the only behavior that is altered, and mice that experience this manipulation show normal anxiety and depressive like behaviors in other tests (open field, tail suspension test and sucrose preference). They show that there is no effect using two types of common inbred strains, Balb/C and C57 mice. As a positive control they show that the chronic social defeat stress procedure, which induces an expected reduction in social interaction, which could also be adaptive except for the fact that the CSDS mice also exhibit anhedonia, arguing that this test has some cross validity for inducing depression. These findings are different than other groups showing that sucrose preference is reduced in the RFSS task, and the authors indicate that is not clear why there is a difference as methodological differences cannot be clearly evaluated. Overall the authors conclude that this is not a good model of depression.

The manuscript is very clearly written and the methods are well described. There is a lot of negative data, which is always more difficult to interpret. My main concern is that their positive control, CSDS was not tested across the full battery of measures (open field, TST, weight gain, locomotor changes), but only the anhedonia test (sucrose preference). It would have been much more compelling to show these tests as affected by the CSDS, so that the lack of effect in the RFSS mice in these tests and in this laboratory would be more clearly due to not being a model of the "depression" construct, instead of an alternate possibility that the OF and TST tests are not set up well for detecting anxiogenic/depression like effects (e.g. floor effects). It is true that these measures have been shown by other groups to be affected by CSDS (at least in susceptible mice), but CSDS can also be inconsistent, and these studies are a case in point that findings by one group (sucrose preference decreases after 5d-RFSS) may not be replicated by another. Whether that means the validity of the test is in question or there are methodological differences is unclear. That is why if the claim is that the RFSS task is not a good model of depression, it is most compelling to offer a solid positive control for each measure to prove that the tests themselves are not flawed. The anhedonia test is the only test that had a direct positive control (a decrease in consumption was detectable in the CSDS mice). Thus one could argue that the overall conclusion should be limited to stating that the 5d-RFSS task is not a test that can model anhedonia, not the more general conclusion that it is not a good model of depression. Because of this lack of positive controls this limitation should be discussed, and the abstract tempered a bit (see below).

Some discussion of the different types of validity that have been tested for the 5d-RFSS task thus far would be helpful in putting this experiment into the context of the greater literature in this task. The use of the word "bona-fide" (which is repeatedly used) for an animal model of depression is a bit strong. I think what the authors are trying to get at is if the 5-day swim procedure has "construct validity" for depression. It is very hard to prove construct validity, but one way is through cross validation, which is being used here, i.e. does the manipulation affect other operational measures of depression in the predicted direction other than simply affecting one measure, immobility. It is important to indicate if the 5-day swim test may have validity in other aspects, e.g. predictive validity for SSRI treatments (this was not specifically addressed in the discussion). This difference in validity types is important because the abstract states "this is not a valid model of human depression", and that is too strong I believe for the data shown here, given the lack of positive controls and no discussion or data that address predictive validity. These issues should be addressed to make the findings and their implications more clear.

Reviewer 2:

n this study, the authors assesses whether five-day repeated forced-swim stress (5d-RFSS) induces a depressive-like behaviour in two mouse strains, C57BL/6J and BALB/cJ showing opposite phenotypes. The manuscript reports that 5d-RFSS did not induce a depressive-like behaviour but an adaptive behavioural response.

The authors are correct this paradigm raises a lot of debate and seems to be more an adaptive learned behavioural response. The forced swim test cannot be considered as a model of depression. This Porsolt test (1977) is the most widely used test of antidepressant action and is also used to infer "depression-like" behaviour, reflecting an expression of "behavioural despair". In any case the scientific community considers the Porsolt test as a model of depression.

Nevertheless, I have major issues of concern with the reported studies.

Major comments:

In all the experiments, the habituation period is missing or too short.

The authors reported just 2 days of habituation to their facility before the onset of experimental treatments. The common habituation is at least 1 week. Why did the authors reduce this crucial period required to obtain solid results? Moreover, they submitted all mice to a stress with the single housing without habituation period. Did they assess the stress level (for example the corticosterone level) of the animal before submitting them to the onset of experiences? In this case, we cannot exclude an experimental bias.

Have the experiments been realized in the home room or a new room? Did the authors offer a habituation period to the new environment/ acclimatization of the testing room to the mice?

In sucrose preference test, animals should be exposed to at least one day with two bottles of water for habituation (Kundakovic et al. 2013). Why didn't the authors choose to submit mice to this habituation?

Another concern is the fact that the authors used different sucrose concentrations. This makes it impossible to clearly compare the results between the two strains. The authors wrote that 1% sucrose solution did not induce sucrose preference in BALB/cJ. However, other studies have already showed a sucrose preference to a 1% sucrose solution in BALB/cJ (Kundakovic 2013, Lewis et al. 2005). To ensure a true comparison, at least the authors should also use 3% in C57BL/6J.

The authors, using the open field test, indicate no center entries in BALB/cJ, suggesting high level of anxiety. How did the mice behave in this anxiety test (freezing, rearing...)? Did mice show stress in this paradigm? Moreover, BALB/cJ travelled half distance compared to C57BL/6J. The authors should discuss this result.

The open-field is a standard measure of response to a novel environment. The missing habituation might be the consequence of the BALB/cJ behaviour.

Moreover, time spent in the inner (center) area of the open field, is a typical measure of anxiety-like behaviour (Prut and Belzung 2003). Did the authors find a difference in this parameter? The authors should provide this data. Although BALB/cJ showed "greater emotionality" compared to the C57BL/6J, they should explore at least once the inner zone.

The arena used in the open field test is unusual, most of the works used a square arena. Please define the criteria to characterize the center zone.

It is confusing that only C57BL/6J mice were exposed to chronic social defeat (CSDS) and that the authors did not use the same behavioral tests to assess the depressive like state in the CSDS and the 5d-RFSS. Did the CSDS affect the body weight? The authors should provide justification. After the CSDS, the mice still show sucrose preference (consumption ˃75%) close to 80%, a sucrose preference considered as strong by the authors, line 262. Is this I suggest discussing these points.

Regarding the statistical analyses, the authors have to carry out repeated-measures ANOVA in Figure 4A, since the same animals have been used to obtain different measures.

Minor comments:

Could the authors give more details regarding the handling (what time, duration, environment....)?

The number of animal used in each procedure is missing except in figure 4 legend. Please correct and indicate everywhere (figure and methods) the "n".

Many times in the manuscript and figures the authors wrote C57Bl/6J and not C57BL/6J. Please correct.
